# 

**Published:** 1906-03-17

**Authors:** 


### NOTICE TO CORRESPONDENTS.

All MSS., Letters, Books for Review, and other matters intended for the Editor should be addressed The Editor, "The Hospital," The Hospital Buildings, 28 & 29 Southampton Street, Strand, W.C.

The Editor cannot undertake to return rejected MS., even when accompanied by stamped directed envelope.

**Notice to Advertisers and Subscribers.**

All Advertisements, Orders for Copies of The Hospital, and Business Communications should be addressed to **THE MANAGER (Not to the Editor),** The Hospital, 28 & 29 Southampton Street, Strand London, W.C.

The Cost of Subscription, post free, is as follows:—Per week, 3½d.; per quarter, 3s. 3d.; per half-year, 6s. 6d.; per annum, 13s. Foreign and Colonial:—Per annum, 19s. 6d., payable, in all cases, in advance.

**_OTICE.—Vol. XXXVIII. of "The Hospital" April to September 1905), in handsome cloth binding, will shortly be on sale at the office of this Journal, price 10s. 6d. Cases for binding the Numbers contained in this Volume 2s. Index to Vol. XXXVIII., 3½d., post free.**

**The Monthly Part for March is now ready. Price 1s., by post, 1s. 3d.**

### BOOKS RECEIVED.

J. Wright and Co.

"Lectures to Teachers on the Prevention of Infectious Diseases." By W. Berry, D.P.H.

Chatto and Windus.

"Herbert Fry's Royal Guide to the London Charities, 1906."

C. Griffin and Co.

"Official Year-book of the Scientific and Learned Societies."

Rebman, Limited.

"Biographic Clinics." Vol. III.

Hodder and Stoughton.

"Practice of Medicine" (Medical Epitome Series).

William Green and Sons.

"The Cleansing, Disinfection, and Protection of the Hands." By Dr. Carl S. Haegler.

J. and A. Churchill.

"Third Treatise on the Effects of Borax and Boric Acid on the Human System." By Dr. Oscar Liebreich.

Great Western Railway.

"The Cornish Riviera."

### Medical Appointments and Vacancies.

### APPOINTMENTS.

Wallis, E. Whishaw, L.D.S., R.C.S. Eng., Assistant Dental Surgeon to the Italian Hospital, Queen Square, W.C.

### VACANCIES.

Birkenhead and Wirral Children's Hospital, Woodchurch Road, Birkenhead.—House Surgeon.

Bolton Infirmary and Dispensary.—Junior House Surgeon.

Bradford Children's Hospital.—House Surgeon.

Bristol, Brislington House Private Asylum.—Assistant Medical Officer.

Brookwood, near Woking, Surrey County Asylum.—Third Assistant Medical Officer.

Burley-in-Wharfedale, West Riding County Council, Scalebor Park.—Assistant Medical Officer.

Carlisle, Cumberland Infirmary.—Resident Medical Officer.

Clayton Hospital and Wakefield General Dispensary.—Junior House Surgeon.

Dudley, Guest Hospital.—Assistant House Surgeon.

Evelina Hospital for Sick Children, Southwark, S.E.—Eight Qualified Clinical Assistants.

Glasgow Eye Infirmary.—Resident Assistant House Surgeon.

Great Northern Central Hospital.—Senior House Physician, Senior House Surgeon, Junior House Physician, and two Junior House Surgeons.

Guy's Hospital.—Two Clinical Assistants in the Ophthalmic Department.

Jerusalem, British Ophthalmic Hospital.—Assistant Surgeon.

King's College (University of London).—Demonstrator of Bacteriology.

King's Lynn, West Norfolk and Lynn Hospital.—House Surgeon.

London Hospital Pathological Institute.—Director.

London Throat Hospital, 204 Great Portland Street, W.—House Surgeon.

Metropolitan Hospital, Kingsland Road, N.E.—House Physician, House Surgeon, Assistant House Physician, and Assistant House Surgeon. Also Casualty Officer.

Middlesbrough, North Ormsby Hospital.—House Surgeon.

Midhurst, King Edward VII. Sanatorium.—Assistant Medical Officer.

Taunton Isolation Hospital.—Medical Attendant.

Westminster Hospital, Broad Sanctuary, S.W.—Casualty Medical Officer.

Winwick, Warrington, Lancashire County Asylum.—Assistant Medical Officer.

### From Probationer to Matron.

### THE SECRET OF SUCCESS IS KNOWLEEGE.

You can acquire knowledge of your Profession only by possessing and studying the best books on the various subjects connected therewith.

### MAY WE SEND YOU

a Catalogue of some of the best and latest Nursing Manuals, from which the following have been selected:—

The Best and Latest Handbook on Midwifery.

### A COMPLETE HANDBOOK OF MIDWIFERY.

By Dr. J. K. Watson, Author of "A Handbook for Nurses." 6/- net, 6/4 post free.

This Hand'ook has been designed to meet the requirements of the Central Midwives Board. It contains a Complete Course of Midwifery, is very fully Illustrated, and is undoubtedly the best Textbook for Nurses entering for the C.M.B. Examinations, as it is only by a thorough knowledge of the subject that success can be ensured.

### SURGICAL INSTRUMENTS AND APPLIANCES USED IN OPERATIONS.

By Harold Burrows, M.B. (Lond.), F.R.C.S., Assistant Surgeon to Bolingbroke Hospital. An Illustrated and Classified List, with Explanatory Notes. Price 1/6 net, 1/8 post free.

### NURSING HINTS TO PROBATIONERS ON PRACTICAL WORK.

By Annesley Voysey. Contains full particulars of a Nurse's duties, and will prove of the greatest assistance to the Probationer Nurse. Price 2/- net, 2/4 post free.

### ELEMENTS OF ANATOMY AND PHYSIOLOGY.

By W. Bernard Secretan, M.D. Illustrated, cloth, 2/- net, 2/3 post free.

"Nurses preparing for an examination will find this book most useful and well worth acquiring."—Guy's Hospital Gazette.

NOW READY.

### LECTURES ON THE NURSING OF INFECTIOUS DISEASES.

By F. J. Woollacott, M.A., M.D., Senior Assistant Medical Officer, Park Hospital, Lewisham. Price 2/6 net, 2/9 post free.

A Matron writes: "I am very pleased indeed to find they are published in book form as I feel sure many nurses will find them most useful. The cost, 2s. 6d., is so little that they are quite within the means of all nurses and Probationers who will do well to study them."

### LECTURES ON HOME NURSING FOR THE POOR.

By a District Nurse. A Series of Lectures based upon the observation of what Teaching on Nursing the Poor most need. A very valuable help to all Nurses engaged in Lecturing or in District Work. Price 1/6 net, 1/8 post free.

### MEDICAL ELECTRICITY AND LIGHT TREATMENT.

By Sister Kate Neale, Guy's Hospital. Illustrated, 2/6 net, 2/9 post free.

The only work on this important subject written especially for Nurses.

### SIMPLE SANITATION: The Practical Application of the Laws of Health to Small Dwellings.

By M. Loane, Superintendent of District Nurses, Portsmouth. Limp cloth, 1/- net, 1/2 post free.

**The Scientific Press, Ltd.,**

Publishers of Nursing Text-Books, Charts, and Case-Books of all Descriptions.

28 & 29 SOUTHAMPTON STREET, STRAND, LONDON, W.C.

### NURSES' UNIFORMS.

### The Nurses' Supply Association,

**9 OXFORD ST. (Opposite Princes' Theatre) MANCHESTER.**

**NURSE' CLOAKS.** In all-wool Cravenette ... from 14/9
In all-wool serge ... ... from 19/6
In good Melton cloth, thoroughly shrunk .... from 21/-
**NURSES COTTON DRESSES.** To measure ... from 10/6
**NURSES' BONNETS.** Prettily trimmed, 5/11, 6/9, 7/3, 8/6
**OUR SP'ECIAL APRON.** Hemstitched, square bib ... 2/4½
**APRONS.** To measure. Circular or square bib, extra stout, linen finished cloth ... ... ... 1/11½
**"SISTER DORA" CAPS.** Fine Scotch lawn 8½d. each.
**CAP STRINGS.** Fine lawn, hemstitched or frilled, 6d. pair.
**STIFF LINEN BELTS.** With tab, 24 to 29½ ins., 11½d. each.
**COTTON BELTS.** Linen finish ... ... 4d. each.
**COLLARS.** Ambulance, 4 fold ... ... 4¾d. each.
**OUR SPECIAL DOUBLE WIDTH ZEPHYR.** Fast colours ... ... ... ... ... 10¾d. yard.
**HERCULES DRILL.** Very strong, fast colours 8¾d. yard.
**DOUBLE WARP ZEPHYRS AND OXFORDS.** Fast colours ... ... ... ... ... ... 7½d. yard.
**IRISH ART LINENS.** In all shades, 36 ins. wide ... ... ... 1/1, 1/2 and 1/5½ yard.
**FINE IRISH APRON LINENS.** 45 ins. wide, from 1/1½ yard.
**OUR CELEBRATED APRON CLOTHS.** 50 ins. wide ... ... ... 7½d. and 10¾d. yard.
**SILK GOSSAMER.** Unspottable Navy, 36 ins. wide, 3/- yard, other colours, 27 ins. wide, 2/- yard.
**SERGES.** All wool, fast dye, wear guaranteed, from 1/4½
**ALPACAS.** Black, navy, grey, myrtle green, from 1/6½ yd.
**BEIGES.** All wool, washable ... ... ... from 2/3½
**CASHMERES.** Fine all-wool ... ... ... 1/11½ yard.

*Illustrated Price List and Patterns free from Dept. H.*

### WILLIAMSON'S SUPERIOR WASHING DRESS FABRICS FOR NURSE'S UNIFORMS

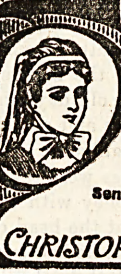

**RELIABLE GOODS AT POPULAR PRICES.**

Haslar and Wynburg Regattas, 7¾d. & 9¾d. yd.

Haircord Gingham, double width, 10¾d. yd.

Oxfords and Indigo Drills, 4¾d. & 6¾d. yd.

Superior Costume Linens, plain colours, 1/1½d. yd.

**Send Postcard for Patterns, mentioning "Hospital."**

CHRISTOPHER WILLIAMSON, 91, EDGWARE RD, LONDON, W.

### DO YOU WANT A GOOD CYCLE?

*Triumphs, Swifts, Premiers, Court Royals, Rovers, Raglans, Singers, Excelsiors, Humbers, Centaurs, &c., &c. Buy a*

### GOOD COVENTRY CYCLE

**AND AVOID RISKS.**

Easiest of Easy Payments. Strictest privacy.

From 5/- Monthly.

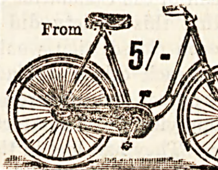

A HIGH-GRADE **COVENTRY CYCLE** With Four Years' Guarantee, **£4 17s. 6d.**

RARE BARGAINS ALSO IN SECOND-HAND MACHINES BY WELL-KNOWN MAKERS.

**SPECIAL TERMS TO NURSES.**

Lists and best advice free on application to Manager—

**IMPERIAL CYCLE SUPPLY Co., COVENTRY.**

